# Associations between Owners’ Reports of Unwanted Ridden Behaviour and In-Hand Behaviour in Horses

**DOI:** 10.3390/ani10122431

**Published:** 2020-12-18

**Authors:** Nicole Romness, Kate Fenner, Jessica McKenzie, Ashley Anzulewicz, Bibiana Burattini, Bethany Wilson, Paul McGreevy

**Affiliations:** 1 Sydney School of Veterinary Science, Faculty of Science, University of Sydney, Camperdown, NSW 2006, Australia; kate@kandooequine.com.au (K.F.); aanz4186@uni.sydney.edu.au (A.A.); bbur2426@uni.sydney.edu.au (B.B.); Bethany.wilson@sydney.edu.au (B.W.); paul.mcgreevy@sydney.edu.au (P.M.); 2 School of Life and Environmental Sciences, Faculty of Science, University of Sydney, Camperdown, NSW 2006, Australia; jmck2527@uni.sydney.edu.au

**Keywords:** conflict behaviour, bolting, rearing, bucking, equitation science, E-BARQ

## Abstract

**Simple Summary:**

Dangerous ridden behaviour in horses, such as bolting, rearing and bucking, are common and may reflect various aspects of the horses’ immediate experience, history and health. They can have a major impact on human safety and horse welfare because of the common misunderstandings of unwelcome behaviour in horses and popular treatments for so-called problem horses. The current study aimed to identify any in-hand behaviours associated with these dangerous ridden behaviours evaluated based on responses (*n* = 1584) to the Equine Behaviour Assessment and Research Questionnaire (E-BARQ). Declining reports of bolting were associated with decreasing problems loading horses onto transporters, increasing social confidence with other horses and other animals, improved leading behaviour and increased tolerance of restraint. Declining reports of rearing were associated with decreasing loading problems, increasing social confidence with other animals and increasing tolerance of restraint. Declining reports of bucking were associated with decreasing loading problems and increasing social confidence with horses and other animals, improved leading behaviour, increasing tolerance of restraint and increasing tolerance of head handling (when bridling/haltering). Findings from the current study could help riders and trainers predict dangerous ridden behaviour before they manifest fully, allowing for remediation that avoids the escalation of force in the training of misunderstood horses and thus improving safety and welfare for both horses and riders.

**Abstract:**

An evidence-based understanding of dangerous or unwelcome behaviour in horses would greatly benefit both horses and humans who interact with them. Using owner-reported data from the Equine Behaviour Assessment and Research Questionnaire (E-BARQ), the current study investigated in-hand behaviours associated with dangerous or unwelcome ridden behaviours, notably bolting, rearing and bucking. Respondents (*n* = 1584) to the ridden horse section of the E-BARQ answered 42 demographic questions, followed by 268 behavioural items. Parallel analysis was conducted to group individual behaviours into rotated components to create independent and dependent indices. Multivariable general linear modelling and ordinal logistic regression were used to identify behaviours associated with bolting, rearing and bucking. Results revealed that safety-from-bolt increased as social confidence with horses (Odds ratio (OR) = 1.06; 95% confidence interval (cf = 1.02–1.09) and other animals (OR = 1.08; cf = 1.03–1.12), compliance in-hand (OR = 1.10; cf = 1.06–1.16) and tolerance of restraint (OR = 1.05; cf = 1.0–1.11) increased; and decreased as loading problems (OR = 0.95; cf = 0.92–0.99) increased. Safety-from-rear increased as tolerance of restraint (OR = 1.07; cf = 1.02–1.12) and social confidence with other animals (OR = 1.05; cf = 1.01–1.09) increased; and decreased as loading problems (OR = 0.94; cf = 0.91–0.98) increased. Safety-from-buck increased as social confidence with horses (b-value = 0.011, *p* < 0.001) and other animals (b-value = 0.010, *p* = 0.002), compliance in-hand (b-value = 0.015, *p* < 0.001), tolerance of restraint (b-value = 0.009, *p* = 0.027) and tolerance of haltering/bridling (b-value = 0.016, *p* = 0.010) increased, and it decreased as loading problems increased (b-value = −0.011, *p* < 0.001). By revealing, for the first time, that specific behaviours on the ground are associated with particular responses in the same horses when ridden, this study advances equitation science considerably. Identification of risk factors for dangerous behaviour while under saddle can improve safety for horses and riders and highlights the importance of effective and humane in-hand training.

## 1. Introduction

Behavioural problems in the ridden horse are common, with their reported prevalence reaching up to 91% (*n* = 1326) in one study of leisure riding horses in the UK [1]. Unwanted behaviours in such high prevalence are likely to contribute to so-called behavioural wastage. When behaviour is sub-optimal, horse and rider safety are jeopardized [2] and horses’ values drop [3,4,5], with an accompanying decline in the care they receive across the four physical domains of welfare: nutrition, environment, health and behaviour [6].

Behavioural problems in the ridden horse have a direct impact on human safety and horse welfare. The rate of human injury due to interactions with horses has been estimated at about 1 per 350–1000 h of horse riding or 18.7 injuries per 100,000 horse-related interactions [7,8,9]. Most reported injuries occur during horse riding, with estimates ranging from 54–79% of horse-related injuries being attributed to being thrown from a horse or falls [7,10]. A horse that is perceived as misbehaving will often be punished for its behaviour in an attempt to correct it. However, punishment, particularly when delivered with excessive force, is more likely to increase fear or stress in the horse and exacerbate the problem than it is to inhibit the behaviour [11,12].

As the debate about the social license to use horses in sport develops [13], the need for ethical equitation is of growing importance [14]. Ethical and effective approaches to behaviour modification involve optimal use of operant conditioning. Operant conditioning in horses largely depends on negative reinforcement whereby aversive stimuli are applied until the horse offers the desired response, at which point the rider or trainer must immediately remove the aversive stimulus. Optimal horse training relies on minimal use of aversive stimuli and the effective management of equine arousal and flight responses [15].

The origins of unwelcome behaviours in horses are complex and multifactorial [15]. They can emerge from a variety of causes that include, but are not limited to, pain, manifestations of a flight response, temperament or hyper-reactivity of the horse, poor communication between horse and trainer, conflicting motivations, poor management, expression of exuberance or a learned (reinforced) behaviour [15,16,17]. Examples can be seen in a suite of counter-predator responses that include bolting, rearing and bucking.

Bolting, rearing and bucking are recognised as clinical signs of pain in the equine vertebral columns [18,19,20,21] with a variety of injuries known to be associated with them. Furthermore, pain has also been shown to be associated with defensive aggression in-hand (i.e., on the ground, when not being ridden), such as threatening to lunge (advance forward towards the handler) or lunging with ears back and threatening to bite or biting [22]. This illustrates how behaviours, with a primary cause of pain, can become learned through negative reinforcement. Current or anticipated pain triggers evasive behaviour by eliciting a response as an attempt to remove or avoid the painful stimuli. If the horse succeeds in removing the stimuli, be that by unseating a rider or making a handler retreat, the behaviour is reinforced. Thus, these dangerous and unwanted behaviours become conditioned responses. As such, they can become ingrained habits that are not easily extinguished. Any primary cause of bolting, rearing, or bucking can be perpetuated through negative reinforcement via this same mechanism. For example, a horse that rears to avoid pain may dislodge its rider and thus terminate the rider’s cues that are associated with pain.

The flight response is a horse’s instinctive behaviour to run away from any real or perceived threats. Bolting, rearing and bucking are considered counter-predator behaviours [23] and may all be observed as a part of an equine flight response [15]. Like responses to pain, these counter-predator behaviours may be reinforced through operant conditioning if the horse is able to escape from the fear-inducing stimulus through expressing one of these behaviours.

In-hand training is an integral step in the process of training horses for ridden and driven work [24]. Through in-hand training, horses first learn to respond to basic cues such as to “go” (accelerate) and “stop” (slow down, stop and step backwards) [25]. Horses are introduced to training through combined reinforcement (positive and negative reinforcement used in combination), and ideally learn to respond to subtle approximations of pressure cues to avoid any need for sustained pressure. To avoid absolute reliance on pressure cues, some trainers apply classical conditioning to associate pressure with other cues, such as an auditory stimulus, to prompt a desired response without the application of any aversive stimulus [26].

Poor timing and inconsistency can lead to unwelcome behaviours. For example, the trainer may fail to release the pressure at the moment the correct response is offered, and the horse may begin to habituate to pressure cues. Such horses may be erroneously diagnosed as lazy or unwilling to learn. It is possible that negative reinforcement deficits in in-hand training may manifest when the same horses are eventually ridden. As ridden horses are first trained in-hand, it is worth considering how unwanted ridden behaviours could be associated with other behaviours when working in-hand. 

Unwanted ridden behaviours are often an expression of confusion or conflicting motivations [16]. Indeed, conflict theory, as described in McLean 2010 [27], proposes that improper application of negative reinforcement can result in stress and confusion that can manifest as occasional hyper-reactive behaviours, also known as conflict behaviours, such as bolting, rearing, or bucking. For example, when a horse is fearful of an object, it will not want to approach it, but if the rider is applying strong acceleratory cues, the horse is conflicted about whether to go forward (in response to the rider’s cues) or backwards (to avoid the object) to the extent that the horse is motivated to trial a hyper-reactive response, such as rearing.

Management factors are associated with higher incidence of bolting, rearing and bucking. Increased locomotor activity, including bucking, often arises with the provision of exercise or turn-out to pasture after periods of confinement which can be characterised as a post-inhibitory rebound effect [28]. Certain behaviours in stabled horses are linked to the expression of undesirable behaviours under-saddle. For example, horses that are aggressive in the stable may be harder to ride than those that are not aggressive animals while unresponsiveness in the stable has been related to reluctance to move forward when ridden [29]. Furthermore, conflict behaviours, such as rearing, bolting and bucking, are associated with use of certain items of equipment (such as whips, spurs) and increased number of riders [30]. Use of stronger cues in a situation where the horse encounters conflicting motivations can escalate the problem by increasing pressure on the horse and thus increasing the likelihood of a hyper-reactive response. Being ridden by multiple different riders could also lead to some loss of stimulus control and higher incidence of conflict behaviours if each rider gives the horse slightly different cues that add to its confusion and contribute to a loss stimulus control. As discussed earlier, conflict behaviour and any primary cause for bolting, rearing and bucking (such as pain) can all be perpetuated and strengthened through reinforcement [15]. For example, the horse that encounters a threat, ignores remedial pressure cues from the rider and bolts home may be rewarded by the renewed presence of familiar conspecifics and the removal of the threat and the pressure cues.

While multiple potential causes for bolting, rearing and bucking have been identified, there is a lack of empirical evidence about which predictive in-hand responses may be associated with unwanted ridden behaviour. The current study was designed to identify which in-hand behaviours and which ridden behaviours, if any, associate with manifestations of conflict. To investigate these potential associations, we used the Equine Behaviour Assessment and Research Questionnaire (E-BARQ) to gather in-hand and ridden behavioural data on domestic horses. The E-BARQ collects detailed owner-reported data on observed behaviours, notably without requiring owners to interpret the horses’ motivations for performing those behaviours. Findings from this study could reveal deficits in handling and riding compliance that are associated with dangerous and unwanted behaviours when ridden. Exposing such relationships could help riders and trainers to predict and mitigate dangerous ridden behaviours before they occur thus improving safety for both horses and riders.

## 2. Materials and Methods

This project was conducted with approval from the University of Sydney Human Research Ethics Committee (approval number: 2012/656). E-BARQ was developed with input from an international panel of professionals in the fields of veterinary science, horse welfare, horse training, elite level competition, equestrian coaching, equitation science and equine behaviour. The questionnaire was piloted and then refined using a rotated component analysis [31]. The questionnaire consists of 97 question matrices covering demographic questions and 215 behavioural items. Respondents were directed to the ridden horse questionnaire if their horse had been ridden in the previous six months but the non-ridden horse questionnaire if their horse had not been ridden in the previous six months.

The questionnaire (see Supplementary File) was created using Qualtrics survey software (Qualtrics Labs Inc, Utah, UT, USA) and was available online at www.e-barq.com. Respondents created an account on the website and were able to save and return to the questionnaire over time, as they wished. Recruitment for the current project was undertaken through use of social media posts and direct e-mail using lists from *Horses and People Magazine* (https://horsesandpeople.com.au/), *Equitation Science International* (https://www.esi-education.com/) and *Kandoo Equine* (https://www.kandooequine.com/). Researchers also distributed the questionnaire through personal networks and publicly in equine interest groups on Facebook. A total of 1584 responses to the ridden survey were used for the current study. The E-BARQ online questionnaire was distributed internationally and drew respondents from 33 different countries. Responses were collected between March and July 2020. Horses comprised of 60% purebred animals representing more than 78 different breeds. Fifty-eight percent were geldings, 38% mares, the remainder being stallions and colts (<1%) and fillies (<2%). Over 80% of respondents reported working with or keeping horses before the age of 16 years, and 83% reported more than 8 years of riding or horse handling experience. Ninety percent of respondents were in the 18 to 64-year age range.

E-BARQ allows for the submission of multiple horses for each owner. For this study, there were 1218 individual users. Seventy-nine percent of users submitted only one horse, 16.3% submitted two horses, and the remaining 4.7% submitted between three and eight horses. For this reason, a linear mixed model approach was not undertaken. The E-BARQ survey has been assessed for intra- and inter-rater reliability and found to be a reliable instrument for assessing behaviour in horses [32].

All questions in the E-BARQ survey were couched for reports specific to only the preceding six months. Response options in the survey were given as a five-point Likert scale from Never to Always with numerical values assigned as follows: Never = 5; Rarely = 4; Sometimes = 3; Usually = 2; Always = 1. The answer choice of “not observed or applicable” was also provided for every question. Where appropriate, some questions asked for responses on a numerical Likert scale from one to five. For example, when respondents were asked to rank the level of aggression or anxiety observed in various situations, they were provided with possible behaviours observed (1 = No signs; 5 = Serious signs (kick, bite, strike).

### 2.1. Trait Selection

#### 2.1.1. Construction of Dependent Indices

Responses from seven E-BARQ items were considered as dependent variables for the three dangerous ridden behaviours of interest: rear, buck and bolt. These seven items used responses from the question in the ridden horse section of E-BARQ, using a 5-point Likert scale to report how often the focal horse was observed to:

- Rear when signalled to go forward on the ground or under saddle [E4_rearforward];
- Rear up when being ridden [E4_rearsaddle];
- Rear up and flip over backwards at any time [E4_ rear_and_flip];
- Buck, pigroot or kick out when signalled to canter when ridden or during groundwork [E12_buck_at_canter];
- Buck at other times than when being signalled to canter (when ridden) [E12_buck_ridden];
- Unseat rider when bucking, pigrooting or kicking out [E12_unseat_rider];
- Bolt (gallop uncontrollably) [EU_bolt].

To combine these into fewer relatively uncorrelated indices, a Parallel Analysis using the Psych package [33] of R statistical software [34] compared the scree of factors of the standardised observed data with those of a random data matrix of the same size and explored underlying number of factors for the seven items. As a result of this parallel analysis, three indices were constructed.

The safety-from-rear index (E4), safety-from-buck index (E12) and safety-from-bolt index (EU) were constructed by assigning a numerical value to scores on the Likert scale of the relevant E-BARQ items and summing these values together. In the case of missing values, the sum was divided by the number of E-BARQ items in the index for which information on that horse was available and multiplied by the number of items used to calculate the index, weighting the missing value according to the horse’s score for similar items, rather than imputing an overall mean. If no E-BARQ items for an index were completed, then a value for that horse was not calculated.

Numerical values were assigned as follows: Never = 5; Rarely = 4; Sometimes = 3; Usually = 2; Always = 1. As both E4 and E12 included information from up to three E-BARQ items, the overall scores for each were between 3 and 15.

Rearing indices other than 15 (indicating horses that never rear) were very rare (<400) across the data set, creating a severe negative skew, that was unlikely to be correctable by simple transformation. For this reason, the rear index was converted to an ordinal index for the purposes of analysis, with score of 15 being low risk, score 12–14 mild elevation, 9–12 moderate elevation and <9 marked elevation.

A high score on each of the three main dependent variables represented desirable behaviour. Bolt was a single ordinal trait, a high score indicating a horse that was less likely to bolt. Rear was an ordinal index, a high score indicating a horse less likely to rear. Buck was a numerical index, a high score indicating a horse less likely to buck.

#### 2.1.2. Determination of Number and Composition of Independent Indices and Other Variables

A total of 29 E-BARQ items, comprising eight rotated components and one non-loading item, from the original E-BARQ development [31] were considered for independent predictor variables. To combine these into fewer relatively uncorrelated indices, after parameterizing the Likert scales of these ordinal items, using the same score assignment as for independent variables (described above), a parallel analysis, comparing the scree of factors of the standardised observed data with that of a random data matrix of the same size, assessed the underlying number of factors (among the 29 items), using the Psych package [33] of R statistical software [34].

Eleven other predictor variables available (including potential confounders) were assessed for potential inclusion in the final model by univariable analysis. Predictors with *p* < 0.2 on univariable analysis (on each of the dependent variable indices constructed in Section 2.1.1 above) were passed into the multivariable model building process for that dependent variable. Safety-from-buck was reversed and log transformed to correct for negative skew and fitted to a univariable model.

### 2.2. Multivariable Model Building

#### 2.2.1. Bolting

Seven explanatory variables were forced simultaneously into the multivariable model. They were Social confidence with other horses, Loading behaviour, Ground handling compliance, Restraint tolerance, Social confidence with other animals, Head-handling tolerance and Social confidence with dogs. Other potential explanatory variables were Country of rider, Age of rider, Horse colour, Horse breed, Rider experience and Horse discipline. Unforced variables were manually added stepwise to an empty model according to *p*-value, admitting Country of rider, Rider experience and Horse breed. Horse discipline was not added, as it appeared to confound with Horse breed, and the residual deviance lowered by only 24.252 for 24 degrees of freedom (thus failing a deviance difference test; *p* = 0.4438). The attempted addition of Age of rider also failed a deviance difference test; *p* = 0.173), as did Horse colour (*p* = 0.073). The forced and unforced models were then combined additively to create the final model.

The final safety-from-bolt model, using an ordinal logistic regression using the ordinal [35] and MASS [36] packages, was as follows:

Safety-from-bolt Index ~ *Country of rider + Experience of rider + Horse breed + Social confidence with other horses + Loading behaviour + Ground compliance + Restraint tolerance + Social confidence with other animals + Head-handling tolerance + Social confidence with dogs.*

Tests for the parallel log odds assumption were performed using the Brant package [37] and the ordinal package [35] (all *p* > 0.05). Surrogate residuals were produced and plotted using the sure package [38].

#### 2.2.2. Rearing

Seven explanatory variables were forced into the multivariable model. They were Social confidence with other horses, Loading behaviour, Ground compliance, Restraint tolerance, Social confidence with other animals, Head-handling tolerance and Social confidence with dogs. Other potential explanatory variables were Gender of Rider, Country of Rider, Age of Rider, Age of Horse, Height of horse, Horse breed and Horse discipline. Unforced variables were added to an empty model according to *p* value, admitting Age of horse, Discipline and Age of rider. Breed was not added as it appeared to confound with Discipline and the model failed a deviance difference test; *p* = 0.4561. Models which added Gender of Rider (*p* = 0.197), Horse height (*p* = 0.190) and Country of rider (*p* = 0.145) also failed to explain significantly more deviance.

The final safety-from-rear model, using an ordinal logistic regression using the ordinal [35] and MASS [36] packages, was as follows:

Safety-from-rear Index ~ *Age of the horse + Horse discipline + Age of the rider + Social confidence with other horses + Loading behaviour + Ground compliance + Restraint tolerance + Social confidence with other animals + Head tolerance + Social confidence with dogs.*

Tests for the parallel log odds assumption were performed using a Brant test using the brant package [37] and likelihood ratio tests of the proportional odds assumption using the ordinal package [35] (all *p* > 0.05). Surrogate residuals were produced and plotted using the sure [38] package.

#### 2.2.3. Bucking

Seven explanatory variables were forced into the multivariable model. They were Social confidence with other horses, Loading behaviour, Ground compliance, Restraint tolerance, Social confidence with other animals, Head tolerance and Social confidence with dogs. Other potential explanatory variables were Age of horse, Horse breed, Horse discipline, Country of rider, Rider experience, Age of rider and Height of horse. Unforced variables were added to an empty model according to p value, admitting Age of horse, Breed, Discipline, Rider country and Experience of rider, with a reduction of Akaike information criterion (AIC) at each addition. Age of rider and Height of horse were not added to the model as this increased AIC.

The final safety-from-buck model was a linear regression as follows:

Safety-from-buck Index ~ *Age of horse + Horse breed + Horse discipline + Horse country + Experience of rider + Social confidence with other horses + Loading behaviour + Ground handling compliance + Restraint tolerance + Social confidence with other animals + Head tolerance + Social confidence with dogs.*

## 3. Results

### 3.1. Trait Selection

#### 3.1.1. Construction of Dependent Indices

The parallel analysis suggested two underlying components. Because bolt did not quite reach a loading of 0.49 when using two factors, the solution for three factors was also examined. Three principal components were extracted, using the psych package and rotated using a varimax rotation, to facilitate interpretation (loading values displayed in Table 1). The three rotated components were related to ridden behaviour so fell under the category of *Equitation (E)* and were labelled as E4 safety-from-rear, E12 safety-from-buck, and EU (E unloaded) safety-from-bolt.

#### 3.1.2. Determination of Number and Composition of Independent Indices

The parallel analysis of independent predictor indices suggested seven underlying components. The E-BARQ question items are shown with their corresponding rotated component classification (see Supplementary File 2: Extracted components) [31]. Seven principal components were extracted using the psych package and rotated using a varimax rotation, to facilitate interpretation. The loadings were as shown in Table 2. 

#### 3.1.3. Other Variables

Analysis of the seven predictor indices and other predictor variables revealed several significant associations with the three dependent indices of safety-from-bolt, safety-from-rear and safety-from-buck. Safety-from-bolt and safety-from-rear were fitted to ordinal logistic regression models and safety-from-buck was fitted to a linear regression model (see Table 3).

### 3.2. Bolting

An increase in the odds of safety-from-bolt was associated with increasing social confidence with horses (OR = 1.06), social confidence with other animals (OR = 1.08), compliance in-hand (*Ground handling compliance*) (OR = 1.10), and tolerance of restraint (OR = 1.05). A higher odds of safety-from-bolt was also associated with variables such as breed in that Warmblood horses had higher odds of safety-from-bolt (OR = 1.89) when compared to crossbreed horses (see Table 4).

A decrease in the odds of safety-from-bolt was associated with increasing loading problems (OR = 0.95). A lower odds of safety-from-bolt was also associated with country of origin in that horses from Belgium (OR = 0.27), South Africa (OR = 0.24), Sweden (OR = 0.20), UK (OR = 0.42), USA (OR = 0.36) and other (OR = 0.26) had decreased odds of safety-from-bolt when compared to the reference country of Australia. Riders with less than 5 years of experience riding were also associated with a lower odds of safety-from-bolt (OR = 0.47) when compared to riders who had ridden horses for their entire life (see Table 4).

### 3.3. Rearing

An increase in the odds of safety-from-rear was associated with tolerance of restraint (OR = 1.05), increasing social confidence with other animals (OR = 1.05) and age (OR = 1.05) (see Table 5). A decrease in the odds of safety-from-rear was associated with an increase in loading problems (OR = 0.94). Lower odds of safety-from-rear was also associated with discipline of the horse in that show-jumpers showed lower odds of safety-from-rear when compared to pleasure riding horses (see Table 5).

Despite no individual level being calculated as significantly different from the reference class, Age of Rider did have an impact on model fit overall. AIC of the unforced model was 2125.336 without Age of Rider included and AIC: 2081.796 with Age of Rider included.

The deviance difference between these two models was 57.54 for 7 degrees of freedom difference which is clearly significant by the chi- square approximation.

It was therefore retained due to our prospectively established model building framework.

### 3.4. Bucking

High scores for safety-from-buck were associated with increasing age (*p* = 0.000), social confidence with horses (*p* = 0.000), social confidence with other animals (*p* = 0.002), compliance in-hand (*p* = 0.000), tolerance of restraint (*p* = 0.027), and tolerance of haltering/bridling (*Head_tolerance*) (*p* = 0.010). Scores for safety-from-buck were also associated with breed in that gaited horses had higher safety-from-buck scores when compared to crossbreed horses (*p* = 0.026). Additionally, riders with less than two years of riding experience showed higher safety-from-buck when compared to riders who had ridden their whole life (*p* = 0.019) (see Table 6).

Low scores for safety-from-buck were associated with increasing loading problems (*p* = 0.000). A lower prevalence of safety-from-buck was also associated with discipline in that companion horses (*p* = 0.005) and pony club horses (*p* = 0.003) showed lower safety-from-buck scores when compared to pleasure riding horses. Additionally, country of origin showed significant results in that horses originating from New Zealand showed low safety-from-buck scores when compared to horses originating from Australia (*p* = 0.009) (see Table 6).

## 4. Discussion

The purpose of the current study was to investigate what in-hand and under-saddle behaviours, as reported by owners in the E-BARQ, were associated with bolting, rearing and bucking in those horses when ridden. Significant risk factors were discovered for all three of these ridden behaviours, and it is worth noting that they are all associated with some combination of problem behaviour in-hand. There was no instance in which desirable in-hand behaviours were associated with unwanted ridden behaviours. This finding highlights the importance of in-hand training.

As previously described by McGreevy et al. 2018 [16], bolting can be observed in horses with a poor response to deceleration cues; bucking can be observed in horses with a poor response to acceleration and deceleration cues, and rearing can be observed in horses with a poor response to deceleration, acceleration and turning cues. It was therefore hypothesized that horses displaying a good response to “go” cues in-hand would be less likely to buck or rear under saddle and horses displaying a good response to “stop” cues in-hand would be less likely to bolt, buck and rear. Findings from this study support these predictions, and the prospect that the three behaviours are independent of each other, as described in further detail below.

Loading problems were associated with increased bolting, rearing and bucking. Loading problems are common, with evidence that loading is a fear-inducing and stressful event for horses that have not been habituated to ramps and enclosed spaces with floors that can tilt under their weight and have not been trained to lead correctly [39]. Instinctively, horses avoid dark, narrow areas, and loading problems can also be exacerbated by a poor response to “go forward” cues, notably from the lead rope or reins [16]. When a horse refuses to load, its stalling behaviour is often reinforced, even if inadvertently, for example, by release of a rope pressure cue [40]. Loading problems may reflect a horse with a heightened flight response (and, as reported here, a tendency to bolt) and/or one that has not been trained to respond effectively to in-hand cues to step forward and therefore is predisposed to trial movement away from the vehicle instead of compliance with the leading cues. In this sense, the current data may support the view that loading problems are often leading problems. Furthermore, rearing has been identified as a conflict behaviour directly associated with loading in that some horses will rear as an expression of conflict when loading is attempted [40]. If this behaviour is successful in helping the horse escape from pressure, it could become a conditioned response which may be trialled in other situations, such as when ridden.

Social confidence with horses was inversely associated with the risk of both bolting and bucking. In this study, social confidence with horses was estimated from, among other traits, the prevalence of aggressive behaviour displayed when near other horses. Aggression can be a learned behavioural response in reaction to either pain or fear. In situations where the horse is unable to escape with a flight response, it may escalate to use aggression. Equally, aggression as a direct result of pain cannot be ruled out because, particularly in cases of chronic pain, it may be a learned avoidance behaviour, that is compounded if horses find fleeing itself painful [22]. Therefore, horses with high scores in this category of E-BARQ may include those that associate other horses with fear or pain. Horses with low scores in this category may show a minimal flight response or associate relatively low level of pain with other horses. Such a reduced flight response and/or relative absence of chronic pain would also fit with the decreased risk of bolting and bucking being observed in these horses. Further studies in this domain should attempt to assess the familiarity of observed horses with neighbouring conspecifics since an established social order may reduce aggression to others.

Social confidence with other animals was inversely associated with the risk of bolting, rearing and bucking. In this study, social confidence with other animals was a direct measure of a horse’s flight response behaviour when in the presence of non-equine animals. Horses that displayed a heightened flight response in these situations may show a heightened flight response to any novel or unhabituated stimuli. As a result, they may also be considered high risk for showing a similar heighted flight response when ridden. These responses could include bolting, rearing or bucking, especially if in the presence of unfamiliar non-equine animals or those it has learned to avoid because of previous aversive encounters.

Tolerance of restraint was inversely associated with the risk of bolting, rearing and bucking. Optimal leading and tying responses typically require a horse to move forward when pressure is applied to the head via a lead rope. That said, untrained horses’ initial reaction may be to pull back in an attempt to escape the pressure. Ideally, horses are conditioned to walk away from the direction of pressure cues from the headcollar via a lead-rope cue [16]. As a result, incorrect application of negative reinforcement, or accidental reinforcement of escape behaviour, such as pulling back until a rope-tie breaks and pressure is released, could both condition undesirable responses to restraint in the horse. Therefore, high tolerance of restraint is a good measure of a horse’s response to “stop” signals from a rope and reflects the effectiveness of prior training. Horses with high scores in this category are presumably those that have been trained effectively in-hand, which could explain the decreased incidence of unwanted ridden behaviour if they have similarly been trained to respond to cues effectively when ridden. That said, the direct transition from head-collar cues in early in-hand training to bit cues in eventual ridden work cannot be assumed since they act on different parts of the head. The current findings suggest great merit in exploring how best to ensure this transition when developing training protocols.

Compliance when led in-hand was inversely associated with the risk of both bolting and bucking. As with tolerance of restraint, in-hand compliance can be interpreted as a measure of the horse’s responsiveness to in-hand cues, in this instance when being led. Horses with high scores in this category are those that readily respond to in-hand cues to “go” and “stop,” reflecting effective training, most commonly by negative reinforcement. These horses also show a decreased risk of bolting and bucking, likely because they have also been trained to respond to “stop” and “go” cues effectively when being ridden. Again, this finding is accompanied by the need to study the optimal means of transferring in-hand cue to ridden cues. Such an investigation may reveal why rearing is not associated with compliance when led in-hand, as one might expect.

Tolerance of head gear (halters and bridles) was inversely associated with the risk of bucking. A low score could be linked to head shyness or could reflect resistance to head restraint. The correlation between bucking and low haltering/bridling tolerance could reflect data on horses that have not been properly habituated to equipment. Another plausible cause of a low score in this category could be any pain in the mouth or poll region that would be exacerbated by placement of a halter or bridle. It is possible this pain could also trigger an escape behaviour response, such as bucking, when ridden. Further research investigating associations between tolerance to restraint, head handling compliance and the types of equipment used would be of interest.

In addition to associations between in-hand and ridden behaviour, the current study also revealed associations between signalment factors and unwanted ridden behaviour. Increasing age was associated with decreased risk of rearing and bucking. As horses age, they become more habituated to various kinds of stimuli that may have previously elicited fear. They have also likely had more time in training both in-hand and when ridden. Additionally, in alignment with the so-called healthy worker effect [41], the horses with chronic pathologies that cause rearing and bucking may have been culled out of the ridden horse population. Therefore, the finding that horses rear and buck less as they age seems logical.

Associations between ridden behaviour and breed or country of origin are difficult to explain. The current findings that increased bolting was associated with several countries of origin (Belgium, South Africa, Sweden, UK, USA, and other) and that increased bucking was associated with horses from New Zealand could reflect the impact of differing riding environments, dependence on housing (that may increase post-inhibitory rebound), training preferences, differences in hyper-reactivity due to genetics or other unidentified factors. More research in this area is needed.

Similarly, Warmblood horses were at increased risk of bolting when compared to crossbreed horses whereas gaited horses were at decreased risk. These findings could reflect breed predispositions (such as the putative result of selecting more for performance than for temperament) or could be attributed to management factors that arise from differing uses for certain breeds. Warmbloods could be more likely to be used in situations where they may experience conflict. For example, they may be away from home and in show-rings and open unfenced areas more often or could also be kept confined more often and fed high concentrate diets as is typical for performance horses. That said, it is worth noting that no significant associations between discipline and bolting were discovered.

Rearing and bucking showed associations with several different ridden disciplines. Show-jumping horses had an increased risk of rearing, and companion horses and Pony Club horses an increased risk of bucking when compared to pleasure riding horses. Show-jumping horses were twice more likely to rear than pleasure riding horses. This difference could be attributed to the more frequent pressure from conflicting motivations that show-jumpers are likely to encounter when compared to pleasure horses. For example, if a show-jumping horse is presented with an especially aversive obstacle, the rider will be cueing the horse to go, while fear of the obstacle will be motivating the horse to stop its advance. Thus, rearing may manifest as a conflict behaviour in this type of situation. As far as bucking is concerned, previous research into the risk factors for ridden misbehaviour in Pony Club horses has identified high-risk individuals as being those with high body condition score, access to pasture with high grass cover, fed supplements, and those not exercised frequently [42]. In contrast to post-inhibitory rebound from confinement seen in competition horses, many of these risk factors could also apply to horses that are primarily used as companion horses.

Overall, the incidence of rearing in the current study was low when compared to bucking and bolting, with fewer than 400 of the 1584 respondents reporting horses that ever reared and the remainder of respondents reporting horses that never reared. While this finding is interesting, further study would be needed to determine the relatively low reported incidence of rearing. Additionally, a larger sample size may help to uncover any additional associations between in-hand behaviour and rearing.

### Limitations

While the survey design was intended to minimise limitations in this study, some merit consideration. Primarily, it must be noted that the cross-sectional nature of this survey means that no conclusions regarding causation can be made. As a self-reported owner questionnaire, there is also the prospect of inherent bias in that different owners may interpret behaviour differently. Attempts were made to write questions that asked about objective and specific observations, but subjective interpretation cannot be completely avoided. The questions in the E-BARQ were limited to behaviour observed solely in the past six months; a decision that was taken in an attempt to improve recall accuracy but one that may have excluded some data. Additionally, while the results were kept anonymous from researchers, the process of signing-up for and completing the E-BARQ did require respondents to provide their name and details. As a result, respondents may have been tempted to provide answers that would show themselves or their horses in a more positive light for fear of being judged or identified. Therefore, the true extent of unwelcome behaviour may have been under-reported. Moreover, some degree of sampling bias may be present in that the demographics of those who completed the E-BARQ may not be representative of the entire target population. The sample of the population who were willing to take the current survey may reflect those with an interest in equitation science or behavioural modification and may not represent the general equestrian population. That said, most E-BARQ respondents were female, which aligns with previous survey-based studies on equitation [43,44].

## 5. Conclusions

This study has revealed in-hand behaviours and ridden behaviours that associate with manifestations of conflict and thus highlights the importance of effective and humane in-hand training. Unwanted behaviours on the ground that are associated with increased reported prevalence of bolting, rearing or bucking include loading problems, low social confidence with horses or other animals, low compliance in-hand, low tolerance of restraint and low compliance with haltering/bridling. When dealing with unfamiliar horses, these behavioural problems in-hand should be checked for and remediated before ridden work commences (or resumes) since they may serve as warnings of dangerous behaviour under-saddle and allow riders to avoid injury.

## Figures and Tables

**Table 1:** Parallel analysis of dependent indices showing loading values with those reaching >0.49 grouped together as three rotated components (RC) and named based on categorization as Equitation (E) related components (values >0.49 displayed in bold).

Dependent Indices	Safety from Buck	Safety from Rear	Safety from Bolt
Rear when signalled to go forward **on the ground or under saddle**	0.07	**0.83**	0.14
Rear up **when being ridden**	0.15	**0.89**	0.04
Rear up and flip over **backwards** at any time	0.22	**0.61**	−0.02
Buck, pigroot, or kick out when signalled to canter **when ridden or during groundwork**	**0.78**	0.12	0.06
Buck at other times **than when being signalled to canter** (when ridden)	**0.82**	0.22	0.11
Unseat rider when bucking, pigrooting or kicking out	**0.83**	0.13	0.13
Bolt (gallop uncontrollably)	0.19	0.09	**0.97**

**Table 2:** Parallel analysis of 29 independent predictor indices with loading values displayed. Values >0.49 were combined to form the seven underlying rotated components (RC) (grouping appear in bold).

E-BARQ Question Item (Rotated Component)	RC1 Social Confidence with Other Animals	RC2 Social Confidence with Other Horses	RC3 Loading Behaviour	RC4 Ground Handling Compliance	RC5 Restraint Tolerance	RC6 Social Confidence with Dogs	RC7 Head Handling Tolerance
Pull when leading at walk (E8)	0.09	0.02	−0.05	**0.85**	0.07	−0.04	0
Pull when leading at trot (E8)	0.08	0.05	−0.05	**0.81**	0.06	0.03	−0.01
Pull when leading and signalled to stop (E8)	0.05	0.05	−0.06	**0.74**	0.08	0.03	0.08
Push handler when offered food (E8)	0.08	0.06	−0.08	**0.49**	0.03	0.03	0.28
Walk into handler when led (E8)	0.01	0.04	−0.11	**0.62**	0.14	0.07	0.16
Throw head up when bridled (E7)	0.08	0.03	−0.05	0.12	0.14	−0.11	**0.74**
Pull back when bridled (E7)	0.08	0.04	−0.07	0.15	0.15	0.04	**0.81**
Pull back when unbridled (E7)	0.06	0.03	−0.09	0.09	0.11	0.15	**0.66**
Load first time onto trailer (E2)	−0.07	−0.08	**0.81**	−0.02	−0.08	0.02	−0.11
Load first time onto truck (E2)	0	−0.07	**0.78**	−0.02	−0.05	0.04	−0.09
Unload slowly off trailer (E2)	−0.04	−0.01	**0.75**	−0.14	−0.1	−0.01	−0.01
Rush off the trailer backwards when loading (E2)	0.1	0.02	**−** **0.68**	0.01	0.15	0	0.09
Unload slowly off the truck (E2)	−0.05	0.03	**0.71**	−0.16	−0.02	−0.08	0.04
Pull back when tied (EU)	0.04	0.01	−0.15	0.1	**0.86**	0.03	0.11
Pull back when tied away from home (T10)	0.1	0.07	−0.1	0.04	**0.88**	0.05	0.13
Not stand still when tied away from home (T10)	0.17	0.08	−0.12	0.28	**0.52**	0.02	0.16
Pull back when tied alone (T10)	0.12	0.03	−0.08	0.09	**0.83**	0.02	0.09
Strongly avoid wild animals (T7)	**0.8**	0.12	−0.06	0.06	0.09	0.08	0.06
Strongly avoid common domestic animals (T7)	**0.8**	−0.02	−0.09	0.13	0.03	0.03	0.04
Strongly avoid uncommon domestic animals (T7)	**0.85**	0.08	−0.04	0.09	0.05	0.06	0.06
Become defensive or aggressive when led towards an unfamiliar horse (T4)	0.06	**0.79**	−0.05	0.07	−0.01	0.13	0.03
Become defensive or aggressive when ridden towards an unfamiliar horse (T4)	0.1	**0.86**	−0.03	0.02	0	0.05	0.02
Become defensive or aggressive when being led beside an unfamiliar horse (T4)	0.02	**0.87**	−0.01	0.02	0.05	0.06	0.04
Become defensive or aggressive when being ridden beside an unfamiliar horse (T4)	0.05	**0.9**	0.02	0	0.05	0.09	0.03
Become defensive or aggressive when being ridden in a group (T4)	0.04	**0.85**	−0.03	0.05	0.09	0.09	0.02
Become defensive or aggressive when being ridden in a group in the arena (T4)	0.05	**0.8**	−0.06	0.09	0.04	0.07	0.01
Become defensive or aggressive when approached by an unfamiliar dog (TU)	0.12	0.22	−0.02	0.05	0.05	**0.86**	0.02
Become defensive or aggressive when approached by a familiar dog (TU)	0.02	0.2	0	0.06	0.05	**0.87**	0.06
Strongly avoid horse drawn vehicles (T1)	**0.64**	0.1	−0.06	0.03	0.23	−0.03	0.09

**Table 3:** The significance of likelihood ratio chi-square (LR χ^2^) statistics for variables associated with incidence of bucking, rearing and bolting, as reported by the owners of 1584 ridden horses (*p* values < 0.05 appear in bold).

Variable	Bolt		Rear		Buck	
	LR χ^2^	*p*-Value	LR χ^2^	*p*-Value	F Value	*p*-Value
Gender of Rider	0.5190	0.9147	8.117	**0.0438**	0.3332	0.8013
Country of Rider	57.076	**<0.001**	16.667	**0.0812**	2.4344	**0.0070**
Age of Rider	14.627	**0.0412**	21.275	**0.0034**	1.554	**0.145**
Laterality of Rider	1.8518	0.3962	2.6184	0.27	0.1865	0.8299
Sex of Horse	2.5015	0.7763	3.0349	0.6946	0.8646	0.4845
Age of Horse	0.0074	0.9316	24.217	**<0.001**	42.21	**<0.001**
Horse colour	18.849	**0.0422**	9.531	0.4826	0.9024	0.5301
Height of Horse	7.6012	0.4734	14.534	**0.0689**	1.4644	**0.1646**
Horse Breed	22.52	**0.0321**	24.434	**0.0177**	3.2479	**<0.001**
Rider of Experience	31.038	**<0.001**	7.5592	0.3731	1.7615	**0.0911**
Horse Discipline	32.7	**0.0364**	43.185	**0.0019**	3.098	**<0.001**

Predictors with *p* < 0.2 on univariate analysis were passed into the multivariate model building process.

**Table 4:** Ordinal logistic regression results: Predictor variables, including the seven created predictor indices (rotated components), found to be associated with safety-from-bolt in owner reported *n* = 1584 horses. Significant values displayed in bold with odds ratio >1 indicating increased safety-from-bolt and odds ratio <1 indicating decreased safety-from-bolt.

Explanatory Variable Level	Odds Ratio	95% Confidence Interval	
**Country of rider (Reference: Australia)**	**1**		
Belgium	**0.27**	**0.08**	**0.87**
Canada	0.62	0.36	1.06
Italy	0.31	0.08	1.17
Mexico	0.30	0.10	0.91
New Zealand	1.35	0.75	2.43
Other	**0.26**	**0.14**	**0.47**
South Africa	**0.24**	**0.08**	**0.68**
Sweden	**0.20**	**0.08**	**0.53**
UK	**0.42**	**0.26**	**0.67**
USA	**0.36**	**0.23**	**0.57**
**Rider Experience (reference: I’ve ridden horses all my life)**	**1**		
No experience with horses	61389.60	61389.57	61389.64
<1 yr experience	1.13	0.21	5.91
<2 yrs experience	0.67	0.23	1.91
<5 yrs experience	**0.47**	**0.27**	**0.83**
<8 yrs experience	0.62	0.33	1.16
>8 yrs experience	0.65	0.42	1.00
Ridden Horses most of my life	0.83	0.57	1.21
**Horse Breed (Reference: Cross Bred)**	**1**		
Arabian	0.62	0.28	1.40
Australian Stock Horse	1.29	0.38	4.37
Standardbred	0.91	0.33	2.57
Thoroughbred	0.77	0.52	1.15
Gaited	1.05	0.34	3.24
Heavy Horse	1.17	0.51	2.72
Iberian	2.05	0.45	9.37
Native	1.07	0.08	13.79
Other	1.04	0.55	1.99
Pony Group	0.92	0.34	2.49
Warmblood	**1.89**	**1.03**	**3.48**
Western/Quarter Horse	1.69	0.93	3.06
**Predictor indices**			
Social confidence with other horses	**1.06**	**1.02**	**1.09**
Loading behaviour	**0.95**	**0.92**	**0.99**
Ground handling compliance	**1.10**	**1.06**	**1.16**
Restraint tolerance	**1.05**	**1.00**	**1.11**
Social confidence with other animals	**1.08**	**1.03**	**1.12**
Head-handling tolerance	1.06	0.97	1.14
Social confidence with dogs	0.94	0.85	1.04

**Table 5:** Ordinal logistic regression results: Predictor variables, including the seven created predictor indices (rotated components), found to be associated with safety-from-rear in owner-reported horses *(n* = 1584). Significant values displayed in bold with Odds Ratio (OR) >1 indicating increased safety-from-rear and OR < 1 indicating decreased safety-from-rear.

Explanatory Variable Level	Odds Ratio	95% Confidence Interval	
Age of horse	1.05	1.02	1.08
Equestrian discipline (reference: Pleasure Horse)			
Adult riding club	0.98	0.43	2.24
Breeding and conformation classes	0.50	0.08	3.16
Companion horse	1.01	0.28	3.56
Competitive riding	0.69	0.31	1.53
Dressage	0.97	0.62	1.53
Endurance	0.57	0.20	1.69
Equitation	2.03	0.43	9.53
Eventing	1.17	0.68	2.03
Liberty	0.48	0.10	2.25
Mounted games	0.95	0.29	3.09
Other	1.46	0.59	3.64
Pony Club	0.53	0.26	1.08
Racing	0.47	0.11	2.05
Show-jumping	**0.52**	**0.31**	**0.87**
Therapy horse	1.15	0.12	11.33
Trail-Riding/Hacking	0.93	0.53	1.65
Western events	0.97	0.48	1.96
Working Equitation	1.19	0.25	5.79
Working cow horse	3.04	0.38	24.56
Age of Rider (Reference: 18–25 years)			
Age 25–34	0.87	0.57	1.33
Age 35–44	1.24	0.78	1.95
Age 45–54	1.31	0.86	2.01
Age 55–64	1.49	0.91	2.43
Age 65–74	2.50	0.99	6.28
Age 75+	0.60	0.04	8.15
Age Under18	0.92	0.48	1.74
Predictor indices			
Social confidence with other horses	1.03	1.00	1.06
Loading behaviour	**0.94**	**0.91**	**0.98**
Ground handling compliance	1.05	1.00	1.09
Restraint tolerance	**1.07**	**1.02**	**1.12**
Social confidence with other animals	**1.05**	**1.01**	**1.09**
Head-handling tolerance	1.00	0.92	1.08
Social confidence with dogs	0.99	0.90	1.09

**Table 6:** Linear regression results: Predictor variables, including the seven created predictor indices (rotated components), found to be associated with safety-from-buck scores from owner-reported data on 1584 horses. Significant values with *p* < 0.05 displayed in bold. Positive estimates indicate increased safety-from-buck and negative estimates indicate decreased safety-from-buck. The coefficient estimates were transformed, and horse age was modelled as a continuous variable. A linear relationship between age and safety-from-buck was determined by visual inspection.

Explanatory Variable Level	Estimate	Std. Error	*t* Value	*p* Value
(Intercept)	−2.507	0.132	−19.029	0.000
Age of the horse	**0.008**	**0.002**	**3.831**	**0.000**
Horse breed (reference: Crossbreed Horse)				
Arabian	−0.051	0.067	−0.761	0.447
Australian Stock Horse	−0.141	0.072	−1.971	0.049
Standardbred	−0.110	0.067	−1.660	0.097
Thoroughbred	−0.035	0.030	−1.177	0.239
Gaited	**0.190**	**0.085**	**2.234**	**0.026**
Heavy Horse	−0.107	0.062	−1.741	0.082
Iberian	0.055	0.077	0.720	0.472
Native	0.204	0.206	0.991	0.322
Other	−0.090	0.048	−1.868	0.062
Pony	0.105	0.066	1.604	0.109
Warmblood	0.045	0.043	1.051	0.294
Western Quarter Horse	0.053	0.043	1.237	0.216
Horse discipline (reference: Pleasure Horse)				
Adult Riding Club	−0.033	0.060	−0.550	0.582
Breeding and conformation showing	**−** **0.321**	**0.158**	**−** **2.025**	**0.043**
Companion horse	**−** **0.249**	**0.089**	**−** **2.800**	**0.005**
Competitive riding	0.038	0.063	0.612	0.541
Dressage	0.026	0.034	0.748	0.454
Endurance	−0.052	0.089	−0.580	0.562
Equitation	0.165	0.093	1.768	0.077
Eventing	0.047	0.040	1.193	0.233
Liberty	0.034	0.125	0.269	0.788
Mounted games	0.132	0.084	1.561	0.119
Other	−0.028	0.059	−0.476	0.634
Pony Club	**−** **0.170**	**0.056**	**−** **3.007**	**0.003**
Racing	−0.101	0.135	−0.745	0.456
Show-jumping	−0.068	0.042	−1.603	0.109
Therapy horse	−0.213	0.159	−1.346	0.179
Trail Riding/hacking	0.005	0.042	0.127	0.899
Western events	0.102	0.058	1.749	0.081
Working Equitation	0.075	0.099	0.755	0.450
Working cow horse	−0.038	0.101	−0.379	0.705
Country of rider (reference: Australia)				
Belgium	−0.170	0.099	−1.711	0.087
Canada	−0.074	0.038	−1.931	0.054
Italy	−0.039	0.119	−0.327	0.744
Mexico	0.089	0.089	0.994	0.320
New Zealand	**−** **0.094**	**0.036**	**−** **2.631**	**0.009**
Other	−0.007	0.046	−0.161	0.872
South Africa	−0.141	0.092	−1.532	0.126
Sweden	−0.035	0.084	−0.413	0.680
UK	−0.086	0.036	−2.402	0.016
US	−0.043	0.035	−1.244	0.214
Rider Experience (reference: I’ve ridden horses all my life)				
No experience with horses	0.236	0.362	0.652	0.515
<1 yr experience	0.218	0.113	1.928	0.054
<2 yrs experience	**0.179**	**0.076**	**2.356**	**0.019**
<5 yrs experience	−0.039	0.044	−0.894	0.372
<8 yrs experience	−0.005	0.048	−0.111	0.912
>8 yrs experience	0.010	0.031	0.335	0.737
Ridden Horses most of my life	−0.002	0.026	−0.083	0.934
Predictor Indices				
Social confidence with other horses	**0.011**	**0.002**	**4.430**	**0.000**
Loading behaviour	**−** **0.011**	**0.003**	**−** **4.022**	**0.000**
Ground handling compliance	**0.015**	**0.003**	**4.387**	**0.000**
Restraint tolerance	**0.009**	**0.004**	**2.213**	**0.027**
Social confidence with other animals	**0.010**	**0.003**	**3.033**	**0.002**
Head-handling tolerance	**0.016**	**0.006**	**2.596**	**0.010**
Social confidence with dogs	−0.003	0.007	−0.478	0.633

## Supplementary Materials

The following are available online at https://www.mdpi.com/2076-2615/10/12/2431/s1. Supplementary File 1—E-BARQ Survey; Supplementary File 2—Extracted components.
